# Loss of Regulator of G Protein Signaling 5 Exacerbates Obesity, Hepatic Steatosis, Inflammation and Insulin Resistance

**DOI:** 10.1371/journal.pone.0030256

**Published:** 2012-01-17

**Authors:** Wei Deng, Xinan Wang, Jinfeng Xiao, Kuoju Chen, Heng Zhou, Difei Shen, Hongliang Li, Qizhu Tang

**Affiliations:** Department of Cardiology, Cardiovascular Research Institute, Wuhan University, Renmin Hospital, Wuhan, China; Northwestern University, United States of America

## Abstract

**Background:**

The effect of regulator of G protein signaling 5 (RGS5) on cardiac hypertrophy, atherosclerosis and angiogenesis has been well demonstrated, but the role in the development of obesity and insulin resistance remains completely unknown. We determined the effect of RGS5 deficiency on obesity, hepatic steatosis, inflammation and insulin resistance in mice fed either a normal-chow diet (NC) or a high-fat diet (HF).

**Methodology/Principal Findings:**

Male, 8-week-old RGS5 knockout (KO) and littermate control mice were fed an NC or an HF for 24 weeks and were phenotyped accordingly. RGS5 KO mice exhibited increased obesity, fat mass and ectopic lipid deposition in the liver compared with littermate control mice, regardless of diet. When fed an HF, RGS5 KO mice had a markedly exacerbated metabolic dysfunction and inflammatory state in the blood serum. Meanwhile, macrophage recruitment and inflammation were increased and these increases were associated with the significant activation of JNK, IκBα and NF-κBp65 in the adipose tissue, liver and skeletal muscle of RGS5 KO mice fed an HF relative to control mice. These exacerbated metabolic dysfunction and inflammation are accompanied with decreased systemic insulin sensitivity in the adipose tissue, liver and skeletal muscle of RGS5 KO mice, reflected by weakened Akt/GSK3β phosphorylation.

**Conclusions/Significance:**

Our data suggest that loss of RGS5 exacerbates HF-induced obesity, hepatic steatosis, inflammation and insulin resistance.

## Introduction

Obesity has reached epidemic proportions not only in Western societies but also in China. It is closely associated with an increase in diseases such as type 2 diabetes, cardiovascular disease and nonalcoholic hepatic steatosis [1], [2], [3], [4]. Hence, understanding the biological basis of obesity-related pathologies, including insulin resistance, dyslipidemia and hyperglycemia, and discovering new therapeutic strategies to restore metabolic function are urgent goals for the biomedical community.

Guanine nucleotide binding proteins (G proteins), including Gαi, Gαs and Gαq family members, are activated by G protein-coupled receptors (GPCRs) signaling from outside of the cell membrane and regulate downstream effectors, such as adenylyl cyclases, phospholipase C, ion channels and cGMP phosphodiesterase [5]. G proteins have been implicated in the regulation of body weight and endocrine/metabolic function, such as the G protein subunit Gαi2 deficiency in adipose tissue and liver of mice produced hyperinsulinemia, impaired glucose tolerance and resistance to insulin, and mice expressing activated Gαs in fat tissue, liver and skeletal muscle displayed normal body mass but blunted glucose metabolism [6], [7].

Regulators of G protein signaling (RGS) proteins are negative regulators of G protein-mediated signaling and act as GTPase accelerating proteins for heterotrimeric G proteins. The effect of downregulating total RGS proteins in obesity has been studied, demonstrating a block in weight gain and insulin resistance in homozygous Gαi2G184S knock-in male mice, which express RGS-insensitive Gαi2 with a G184S mutation that blocks RGS protein binding and GTPase acceleration, fed an HF [8]. Mice without RGS2, which is known to limit signals mediated via Gαs- and Gαq-coupled GPCRs, exhibited greatly reduced fat deposits, decreased serum lipids, low leptin levels, improved glucose clearance and insulin sensitivity [9]. Functional diversity may exist among RGS proteins and RGS5 is an important member of the RGS protein superfamily that has recently been reported to regulate cardiac hypertrophy, atherosclerosis and angiogenesis through the inhibition of several Gαi- and Gαq-mediated signaling pathways [10], [11], [12], [13]. However, the role of RGS5 in obesity and associated metabolic disorders is not clear.

We hypothesized that the absence of RGS5 should modify the development of obesity and alter metabolic state during an HF and used RGS5 knockout (KO) and wild-type (WT) mice to demonstrate this hypothesis. This study is the first to clarify the role of RGS5 in obesity-associated metabolic dysfunction and insulin sensitivity.

## Materials and Methods

### Mouse experiments

All animal procedures were performed in accordance with the Guide for the Care and Use of Laboratory Animals, published by the US National Institutes of Health (NIH Publication No. 85-23, revised 1996) and were approved by the Institutional Animal Care and Use Committee at Renmin Hospital of Wuhan University, China (protocol 00012012).

RGS5 KO mice on a C57BL/6 background were generated by methods described previously [10]. Male RGS5 KO mice and their C57BL/6 littermates were exposed to a 12-h light/12-h dark schedule and maintained at a constant temperature of 22°C. Prior to 8 weeks of age, all mice were fed standard laboratory chow and water ad libitum, and then mice were randomly allocated to either a normal chow (10 kcal% fat, D12450B, Research Diets) or a high-fat diet (60 kcal% fat, D12492, Research Diets) for 24 weeks. In this study, all investigations were completed in a fasted state, with food removed for 6 hours (from 8AM to 2PM) and free access to water. Feed consumption was measured weekly, and body weight and plasma glucose were measured every four weeks. Blood samples were collected via a retro-orbital bleed every eight weeks, and epididymal adipose, liver and gastrocnemius muscle samples were harvested after 24 weeks of diet. All blood and tissue samples for biochemical analysis were stored at −80°C.

### Plasma determinations

Plasma glucose was determined using a glucometer via tail vein blood sampling (OneTouch UltraEasy, LifeScan). Blood insulin, lipid and adipokines were measured by ELISA.

### Glucose tolerance tests (GTT) and insulin tolerance tests (ITT)

Mice were fasted for 6 hours with free access to water and subsequently received an intraperitoneal injection of glucose (1 g/kg; Sigma-Aldrich Co., St. Louis, MO, USA) or insulin (0.75 units/kg; Novolin R, Novo Nordisk Co., Bagsvaerd, Denmark). Blood samples were taken from the tail vein prior to injection and at 15, 30, 60 and 120 minutes after injection. Blood glucose was determined using a glucometer, as described above.

### Histological analysis and morphometry

A portion of epididymal adipose tissue and liver was fixed overnight in 10% formalin, dehydrated in an ethanol bath, and paraffin-embedded. Sections were cut and then stained with hematoxylin-eosin for the observation of adipose and liver tissue structure (BX51, Olympus). A portion of Liver was frozen sectioned and stained for Oil Red O. Single adipocyte was measured using an image quantitative digital analysis system (Image-Pro plus 6.0); one hundred adipocytes in the epididymal adipose tissue were measured in each group.

### RNA extractions and real-time PCR

Total RNA was isolated using the TRIzol reagent (Invitrogen) from various organs, and quantitative RT-PCR analysis was performed using the LightCycler 480 SYBR Green 1 Master Mix (Roche) and the LightCycler 480 QPCR System (Roche). Target gene mRNA expression was normalized to the internal control β-actin and expressed relative to the control group.

### Western blot analysis

Protein samples denatured in SDS sample buffer (125 mmol/l Tris-HCl, pH 6.8, 50% glycerol, 2% SDS, 5% mercaptoethanol, and 0.01% bromophenol blue) were subjected to SDS-PAGE and blotted onto Immobilon-FL transfer membrane (Millipore). Blotted membranes were blocked with 5% skim milk in Tris-buffered saline containing 0.1% Tween 20 for 2 hours and were subsequently incubated with primary antibodies against JNK, NF-κBp65, IκBα, Akt, GSK3β, phospho-JNK (Thr183/Tyr185), phospho-IκBα (Ser32/36), phospho-NF-κBp65 (Ser536), phospho-Akt (Ser473) and phospho-GSK3β (Ser9) proteins (Cell Signaling) overnight at 4°C. After three washes in Tris-buffered saline containing 0.1% Tween 20, the membranes were incubated with anti-mouse or anti-rabbit IgG for 1 hour. Quantification of western blots was performed by the Odyssey infrared imaging system (Li-Cor Biosciences) to detect protein expression. The specific protein expression levels were normalized to GAPDH for the total cell lysate.

### Statistics

Data are expressed as means ± SEM. Differences among groups were determined by two-way ANOVA followed by a Tukey's post-hoc test. Comparisons between two groups were performed using an unpaired Student's *t*-test. A value of *p*<0.05 was considered significant.

## Results

### RGS5 deficiency markedly increased body weight, fat mass and adipocyte size

**Figure 1:**
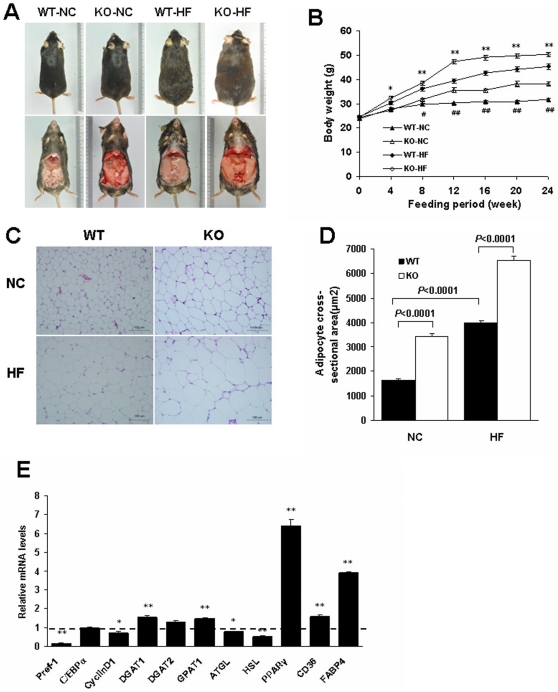
Effect of RGS5 deficiency on body weight, fat mass and adipocyte size. **A** Representative photograph of WT and KO mice fed an NC and HF for 24 weeks. **B** Growth curves of WT (black) and KO (blank) mice fed an NC (triangle) and an HF (rhombus) (n = 8–11). **C** Histology of visceral adipose tissue with hematoxylin-eosin. **D** Mean adipocyte size of the visceral adipose tissue in WT (black) and KO (blank) mice fed an NC and an HF for 24 weeks (n = 5). **E** Expression levels of mRNA related to adipogenesis in the visceral fat of WT (a dotted line at value 1) and KO (black) mice fed an HF for 24 weeks (n = 6). Values represent means ± SEM. #*p*<0.05 and ##*p*<0.01 compared with WT mice fed an NC; *p<0.05 and **p<0.01 compared with WT mice fed an HF.

The initial weights of mice ranged from 23.5 to 25.0 g with no difference among the groups (Table 1). First, we demonstrated that RGS5 KO mice experienced an increase in body mass relative to littermate controls during the feeding period from 4 to 24 weeks with an HF and from 8 to 24 weeks with an NC; these body mass increases were associated with an increase in food intake (Fig. 1A, B and Table 1). Additionally, the RGS5 KO mice had significantly increased fat mass and liver weight at 24 weeks of feeding (Table 1).

**Table 1:** Effect of RGS5 deficiency on body weight at initial of feeding and body weight, mean food intake, fat and liver weight after 24 weeks of feeding (n = 8–10).

*Variable*	*NC*		*HFD*	
	WT	KO	WT	KO
**Body weight (initial) (g)**	23.82±0.19	24.41±0.32	24.74±0.32	24.39±0.31
**Body weight (24 weeks) (g)**	31.86±0.57	38.33±0.83##	45.24±1.11##	50.28±0.99**
**Visceral fat weight (g)**	1.24±0.09	3.10±0.24##	3.68±0.18##	4.97±0.27**
**% Body weight**	3.89±0.27	8.03±0.49##	8.10±0.22##	9.89±0.49*
**Liver weight (g)**	1.34±0.04	1.64±0.08##	1.87±0.11##	2.54±0.09**
**% Body weight**	4.20±0.11	4.30±0.22	4.12±0.19	5.05±0.17**
**Food intake(kcal/mice/week)**	72.62±2.81	85.63±3.45##	88.54±3.42##	95.90±3.73

Data are means ± SEM.

## *p*<0.01 vs. WT mice fed an NC;

*p<0.05 and

**p<0.01 vs. WT mice fed an HF.

The quantity of visceral adipose tissue is the major driver of adipose tissue function, and two factors contribute to fat deposition: the increased size of existing adipocytes because of fat accumulation and the formation of new adipocytes through adipogenesis [14]. To determine whether the increase in fat mass was due to increased adipocyte cell size and/or number, we assessed both and focused on the expression of key genes associated with adipogenesis in epididymal adipose tissue of mice fed an HF. RGS5 KO mice displayed hypertrophic adipocytes relative to the controls (Fig. 1C, D). There was reduced expression of several anti-adipogenic factors, such as Preadipocyte factor-1 (Pref-1) and cyclin D1, and markedly elevated expression of adipogenesis genes, including PPARγ, Cluster of differentiation 36 (CD36) and Fatty acid-binding protein 4 (FABP4), in the fat of KO mice compared with that of their controls; however, there was no difference in CCAAT/enhancer-binding protein α (C/EBPα) expression between the two groups (Fig. 1E). The expression of Diacylglycerol acyltransferase 1 (DGAT1), DGAT2 and Glycerol-3- phosphate acyltransferase 1 (GPAT1) was elevated or exhibited a higher tendency; however, in contrast with lipogenesis, the expression levels of the rate-limiting enzymes of lipolysis, Adipose triglyceride lipase (ATGL) and Hormone-sensitive lipase (HSL), were decreased in the adipose tissue of KO mice (Fig. 1E).

### RGS5 deficiency exacerbated metabolic dysfunction and inflammation in serum

**Figure 2:**
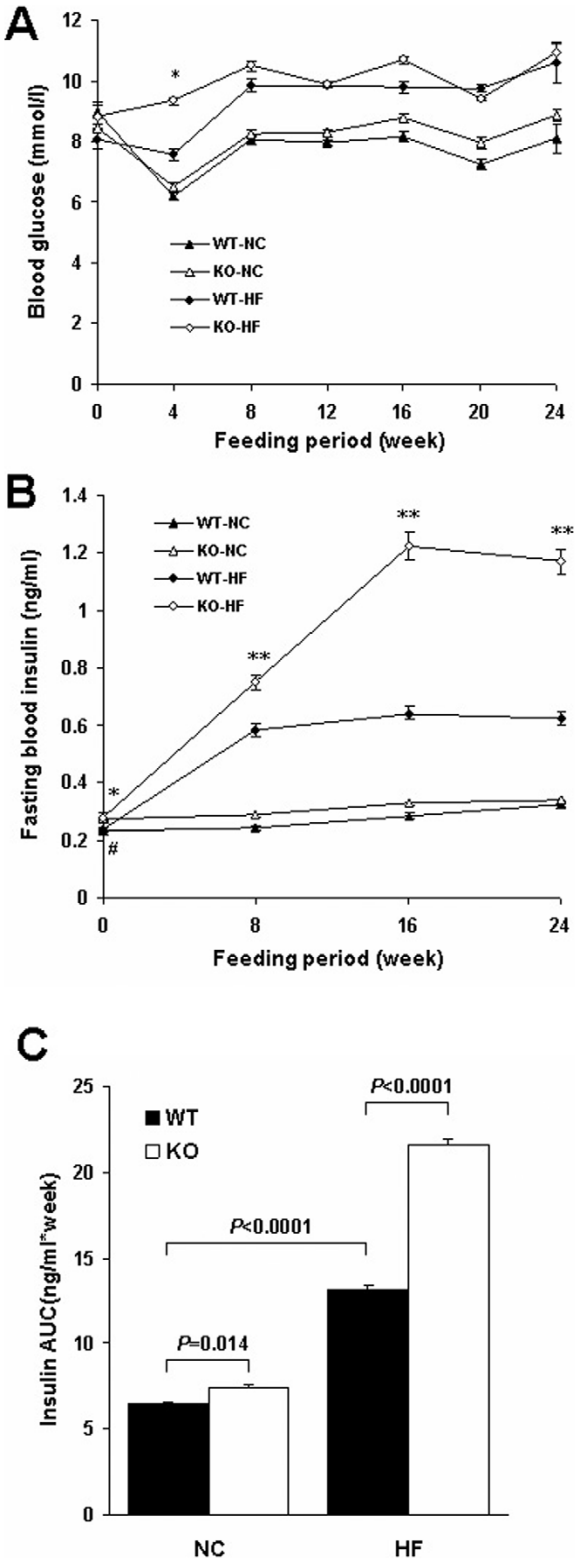
RGS5 deficiency exacerbated hyperinsulinemia. **A** The level of fasting blood glucose in WT (black) and KO (blank) mice fed an NC (triangle) and HF (rhombus) from 0 to 24 weeks (n = 8–11). **B,C** The level of plasma insulin in WT (black) and KO (blank) mice fed an NC(triangle) and HF (rhombus) from 0 to 24 weeks (B) and corresponding AUC (C) (n = 8). Values represent means ± SEM. #*p*<0.05 and ##*p*<0.01 compared with WT mice fed an NC; *p<0.05 and **p<0.01 compared with WT mice fed an HF.

We investigated whether blood glucose, blood insulin, circulating metabolism and inflammation state in obese mice were exacerbated by RGS5 deletion. Although there was no difference in fasting glucose levels, RGS5 KO mice fed an HF exhibited a markedly unremitting elevation in plasma insulin compared with the control mice fed an HF (Fig. 2A, B). At initial of feeding, the markers of inflammation and lipid profiles were no difference among the groups except FFA (Table 2); However, after 24 weeks of feeding, fasting plasma triacylglycerol, cholesterol and LDL levels were significantly higher in RGS5 KO mice than in WT mice fed an HF but were not distinguishable between genotypes in mice fed an NC (Table 3). Fasting plasma FFA levels were markedly higher and HDL levels were lower in RGS5 KO mice than in the controls, regardless of diet (Table 3). Circulating leptin and resistin levels were increased and adiponectin levels were lower in RGS5 KO mice compared with littermate controls fed an HF, whereas these levels were not distinguishable between genotypes in mice fed an NC (Table 3). Circulating inflammatory factors, including IL-1β, IL-2, IL-6, TNFα and MCP-1, were elevated in RGS5 KO mice fed an HF compared with controls (Table 3).

**Table 2:** Metabolic and inflammatory variables in circulation at initial of feeding (n = 8).

*Variable*	*NC*		*HFD*	
	WT	KO	WT	KO
**FFA (mmol/l)**	0.70±0.03	0.93±0.04##	0.77±0.04	0.95±0.04**
**TG (mg/dl)**	38.93±1.92	41.50±1.73	36.48±1.46	40.93±1.71
**TC (mg/dl)**	129.40±6.99	133.81±5.63	133.11±5.77	135.21±5.69
**HDL (mg/dl)**	94.56±3.34	98.52±4.14	98.40±4.90	97.24±4.09
**LDL (mg/dl)**	17.67±0.64	19.30±0.80	17.67±0.53	19.51±0.80
**leptin (pg/ml)**	4185.08±133.44	4272.40±179.24	4062.63±179.68	4261.14±180.03
**resistin (pg/ml)**	878.84±42.93	901.80±37.38	779.53±29.21	887.01±37.42
**Adiponectin (ug/ml/g)**	1.18±0.06	1.31±0.05	1.17±0.05	1.27±0.05
**IL-1β (pg/ml)**	37.16±1.60	42.40±1.77	40.18±1.65	40.83±1.68
**IL-2 (pg/ml)**	11.62±0.63	10.80±0.44	10.98±0.52	11.31±0.45
**IL-6 (pg/ml)**	0.67±0.03	0.71±0.03	0.70±0.03	0.67±0.02
**TNF-α (pg/ml)**	2.21±0.10	2.14±0.09	2.33±0.08	2.25±0.08
**MCP-1 (pg/ml)**	21.24±0.66	23.10±0.96	23.06±1.03	22.72±0.93

Data are means ± SEM.

## *p*<0.01 vs. WT mice fed an NC;

**p<0.01 vs. WT mice fed an HF.

**Table 3:** Metabolic and inflammatory variables in circulation after 24 weeks of feeding (n = 8).

*Variable*	*NC*		*HFD*	
	WT	KO	WT	KO
**FFA (mmol/l)**	0.77±0.03	0.94±0.03##	1.12±0.05##	1.55±0.05**
**TG (mg/dl)**	41.35±1.72	44.60±1.86	50.54±2.10##	64.19±2.69**
**TC (mg/dl)**	135.30±5.69	145.19±6.08	255.19±10.76##	342.35±14.12**
**HDL (mg/dl)**	89.50±3.75	74.72±3.12##	74.20±3.11##	55.23±2.30**
**LDL (mg/dl)**	17.93±0.72	19.18±0.80	24.53±1.00##	32.88±1.33**
**leptin (pg/ml)**	5351.95±225.92	5531.80±233.29	12442.90±525.18##	16355.70±690.77**
**resistin (pg/ml)**	922.48±38.68	971.84±40.81	1310.94±54.49##	1552.38±65.34**
**Adiponectin (ug/ml/g)**	1.13±0.04	1.02±0.04#	0.57±0.02##	0.34±0.01**
**IL-1β (pg/ml)**	39.96±1.63	44.52±1.86	57.88±2.27##	69.86±2.86**
**IL-2 (pg/ml)**	13.95±0.55	14.61±0.40	17.29±0.72##	19.19±0.79*
**IL-6 (pg/ml)**	0.73±0.02	0.92±0.04##	1.21±0.03##	1.66±0.07**
**TNF-α (pg/ml)**	2.88±0.10	2.76±0.10	3.66±0.12##	5.13±0.21**
**MCP-1 (pg/ml)**	24.21±0.99	22.55±0.70	29.68±1.17##	39.59±1.65**

Data are means ± SEM.

# *p*<0.05 and

## *p*<0.01 vs. WT mice fed an NC;

*p<0.05 and

**p<0.01 vs. WT mice fed an HF.

### RGS5 deficiency exacerbated hepatic steatosis

**Figure 3:**
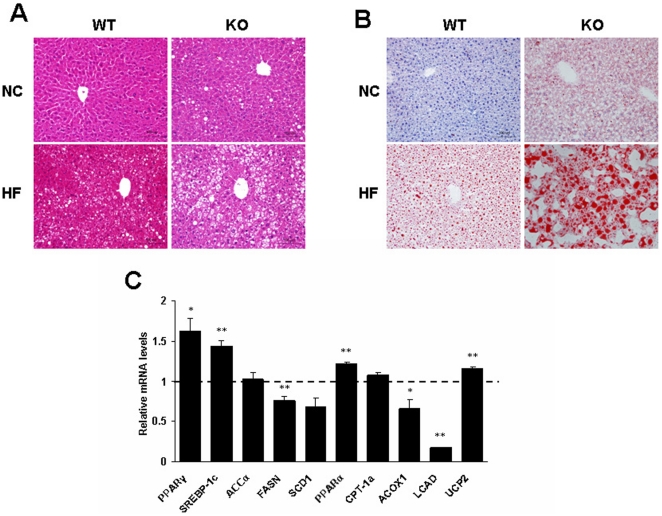
RGS5 deficiency exacerbated fat accumulation in liver. **A** Histology of liver with hematoxylin-eosin. **B** Histology of liver with oil red O. **C** Expression levels of mRNA related to fatty acid metabolism in the liver of WT (a dotted line at value 1) and KO (black) mice fed an HF for 24 weeks (n = 6). Values represent means ± SEM. *p<0.05 and **p<0.01 compared with WT mice.

Next, we tested the association between increased liver weight and hepatic steatosis. Histological analysis revealed that RGS5 KO mice showed a significantly increased accumulation of hepatic lipid droplets, as compared with WT littermates (Fig. 3A, B). When fed an HF, the levels of the mRNAs for PPARγ and Sterol regulatory element-binding protein 1c (SREBP-1c), transcription factors that regulate the expression of genes important in lipid synthesis, were markedly increased in the livers of RGS5 KO mice, with no changes in the expression levels of Acetyl-CoA carboxylase α (ACCα) and Stearoyl-CoA desaturase 1 (SCD1) and decreased expression levels of Fatty acid synthetase (FASN) (Fig. 3C). The expression level of Carnitine palmitoyltransferase- 1a (CPT-1a), which is involved in fatty acid oxidation, was not affected by RGS5 deletion, whereas Acyl-CoA oxidase 1 (ACOX1) and Long-chain acyl-CoA dehydrogenase (LCAD) were decreased in RGS5 KO obese mice (Fig. 3C).

### RGS5 deficiency increased fatty acid oxidation in adipose tissue and skeletal muscle

**Figure 4:**
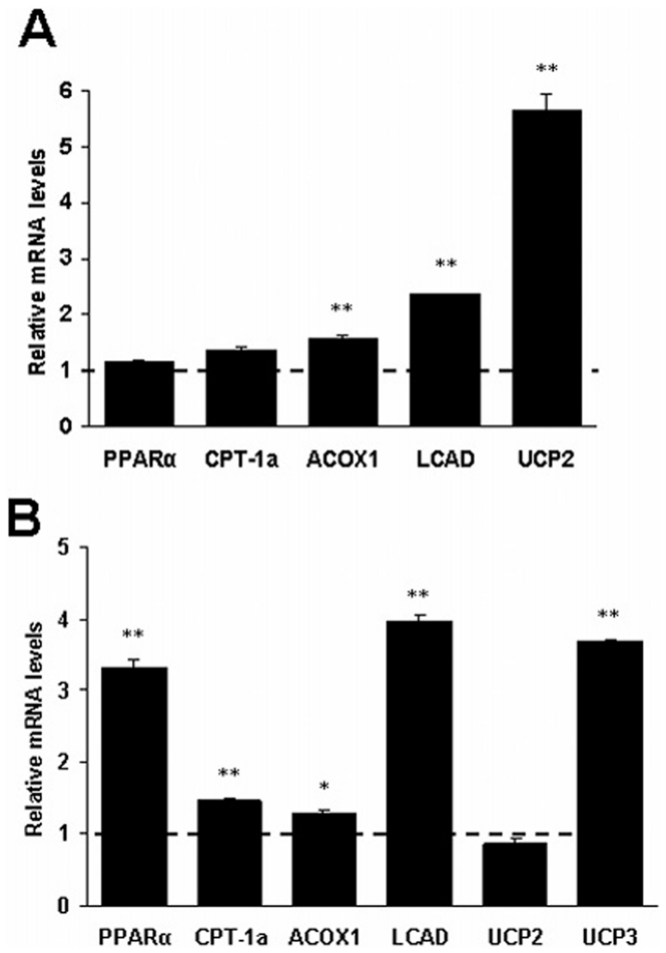
RGS5 deficiency increased fatty acid oxidation in the adipose tissue and skeletal muscle of mice fed an HF for 24 weeks. **A** Expression levels of mRNA related to fatty acid oxidation in the visceral adipose tissue of WT (a dotted line at value 1) and KO (black) mice (n = 6). **B** Expression levels of mRNA related to fatty acid oxidation in the gastrocnemius muscle of WT (a dotted line at value 1) and KO (black) mice (n = 6). Values represent means ± SEM. *p<0.05 and **p<0.01 compared with WT mice.

To investigate whether the energy consumption state is influenced by RGS5 deletion in obese mice, we also examined related enzymes associated with fatty acid oxidation in the epididymal adipose tissue and gastrocnemius muscle. We observed that the mRNA levels of PPARα and the PPARα-regulated genes CPT-1a, ACOX1, LCAD, Uncoupling protein 2 (UCP2) and UCP3 were elevated or exhibited an increasing trend in the adipose tissue, as well the mRNA expression of PPARα, CPT-1a, ACOX1, LCAD and UCP3 were markedly increased in the skeletal muscle (Fig. 4A, B).

### RGS5 deficiency was associated with increased macrophage recruitment and inflammation in adipose tissue, liver and skeletal muscle

**Figure 5:**
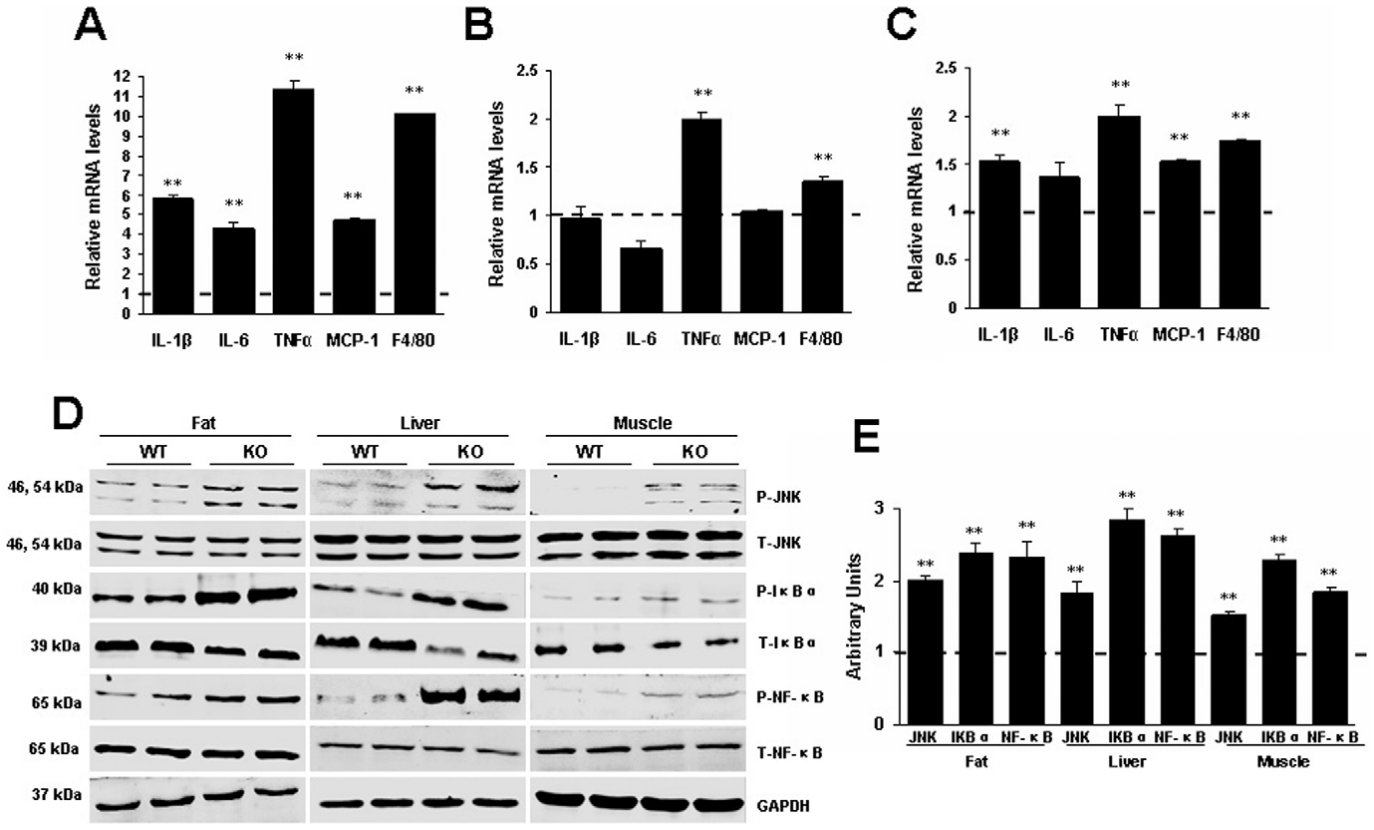
RGS5 deficiency promoted inflammation and JNK, NF-κB signaling in the adipose tissue, liver and skeletal muscle of mice fed an HF for 24 weeks. **A** Expression levels of mRNA related to inflammation in the visceral adipose tissue of WT (a dotted line at value 1) and KO (black) mice (n = 6). **B** Expression levels of mRNA related to inflammation in the liver of WT (a dotted line at value 1) and KO (black) mice(n = 6). **C** Expression levels of mRNA related to inflammation in the gastrocnemius muscle of WT (a dotted line at value 1) and KO (black) mice (n = 6). **D** Phosphorylation levels of proteins related to inflammatory signaling in the visceral adipose tissue, liver and gastrocnemius muscle of KO and WT mice (n = 6). **E** Quantitative measurements of p-JNK, p-IκBα and p-NF-κBp65 protein relative to their total protein between WT mice (a dotted line at value 1) and KO mice (black). Values represent means ± SEM. *p<0.05 and **p<0.01 compared with WT mice.

Inflammation is a key component of obesity-associated insulin resistance. To examine the association of the *Rgs5* gene and inflammation with obesity, we investigated the gene expression of inflammatory mediators in major insulin-responsive tissues of mice fed an HF. We found that the expression levels of F4/80, which is a specific marker of mature macrophages, and TNFα were significantly elevated in the epididymal adipose, liver and skeletal muscle of KO mice compared with those of WT mice (Fig. 5A, B, C). The levels of IL-1β, IL-6 and MCP-1 mRNA were increased in the adipose and skeletal muscle (Fig. 5A, B, C). These results implied an increase in the recruitment of macrophages and the infiltration of inflammation into these tissues of KO mice. JNK and NF-κB play pivotal roles in inflammation induced by obesity, and phosphorylation of IκBα is essential for release of active NF-κB. In accordance with the presence of these elevated inflammatory mediators, we observed elevated phosphorylation levels of JNK, IκBα and NF-κBp65 proteins in the adipose tissue, liver and skeletal muscle of RGS5 KO mice (Fig. 5D, E).

### RGS5 deficiency produced decreased peripheral insulin sensitivity

**Figure 6:**
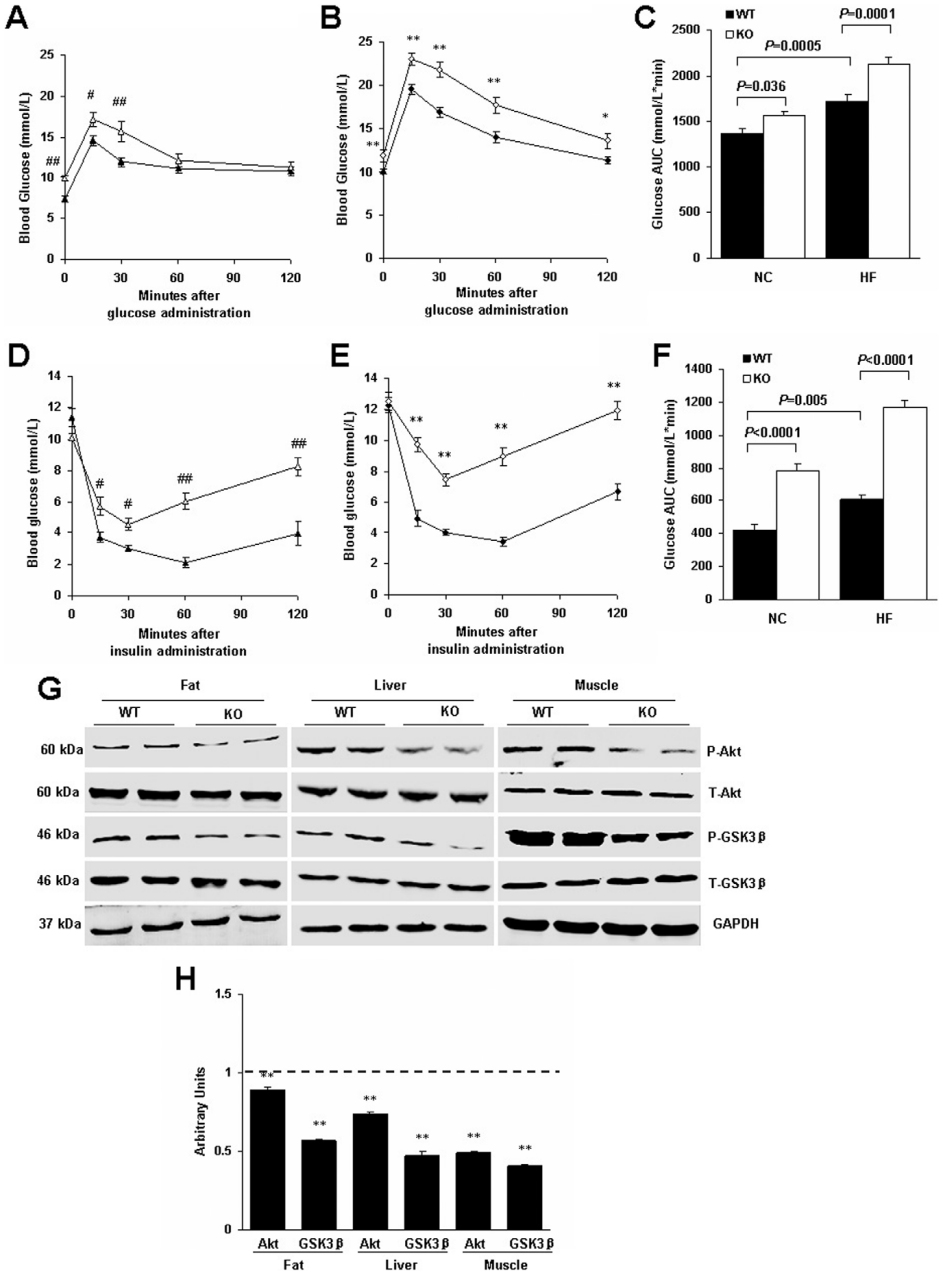
RGS5 deletion resulted in decreased peripheral insulin sensitivity and suppressed the Akt/GSK3β signaling pathway in mice fed an HF for 24 weeks. **A,B,C** Blood glucose levels during GTT were determined at the indicated time points after i.p. injection with a bolus of glucose in WT and KO mice fed an NC (A) and HF (B) and corresponding AUC (C) (n = 6–8). **D,E,F** Blood glucose levels during ITT were determined at the indicated time points after i.p. injection with a bolus of insulin in WT and KO mice fed an NC (D) and HF (E) and corresponding AUC (F) (n = 6–8). **G** Phosphorylation levels of proteins related to insulin signaling in the visceral adipose tissue, liver and gastrocnemius muscle of WT and KO mice (n = 6). **H** Quantitative measurements of p-Akt and p-GSK3β protein relative to their total protein between WT mice (a dotted line at value 1) and KO mice (black). Values represent means ± SEM. #*p*<0.05 and ##*p*<0.01 compared with WT mice fed an NC; *p<0.05 and **p<0.01 compared with WT mice fed an HF.

Type 2 diabetes is caused by a combination of insulin resistance in the adipose tissue, liver and skeletal muscle and impaired insulin secretion from the pancreatic islets [15]. Next, we determined whether RGS5 plays a role in mediating obesity-induced insulin resistance. Following 24 weeks of feeding, RGS5 KO mice showed a significant impairment of glucose tolerance compared with the control mice, regardless of diet; furthermore, the glucose AUC during GTT was increased (Fig. 6A, B, C). Additionally, glucose concentrations after insulin challenge and the glucose AUC during ITT were increased by both NC and HF treatment (Fig. 6D, E, F). The phosphorylated levels of Akt/GSK3β, critical molecules in the insulin signaling pathway, were reduced in the visceral adipose tissue, liver and gastrocnemius muscle of RGS5 KO mice compared with the control mice fed an HF (Fig. 6G, H).

## Discussion

Abnormal lipid metabolism and inflammation are widely recognized as the most important factors in the etiology of obesity-induced whole-body insulin resistance and metabolic diseases. The present study used a diet-induced obesity paradigm with RGS5 KO and WT mice. The major findings are as follows: (i) RGS5 deletion increases dyslipidemia, obesity, adipose tissue deposition, hepatic steatosis and circulating inflammation; (ii) RGS5 deletion exacerbates inflammation and activates the JNK and NF-κB inflammatory signaling pathways in the adipose tissue, liver and skeletal muscle when mice are fed an HF; (iii) RGS5 deficiency results in decreased systemic insulin sensitivity accompanied by weakened Akt/GSK3β signaling in the adipose tissue, liver and skeletal muscle when mice are fed an HF. These findings demonstrate that RGS5 plays a novel and indispensable role in regulating obesity and insulin sensitivity.

Many Gαi- coupled receptors have the anti-lipolytic ability through Gαi-protein mediated inhibition of adenylyl cyclase activation and cAMP accumulation in adipose tissue and elevated cAMP level can increase lipolysis [16], [17]. RGS5 deficiency would likely involve enhanced Gαi-mediated signal pathway and be in charge of increased lipogenesis (mediated by DGAT1, DGAT2 and GPAT1) and decreased lipolysis (mediated by HSL and ATGL), under the conditions of hyperinsulinemia and high FFA, which can induce adipocyte hypertrophy [18], [19], [20]. The high FFA concentration may result from an impaired buffering capacity for lipid storage in the postprandial state due to the consequent prolonged absorption time rather than the increased fasting adipocyte lipolysis when RGS5 KO mice are compared with controls [21]. In addition, increased adiposity is often characteristic of an increase in the number of adipocytes. Accordingly, RGS5 deletion suppressed the expression of anti-adipogenic factors, such as Pref-1 and cyclin D1. Pref-1, which is expressed in preadipocytes, is an inhibitor of adipocyte differentiation, and its overexpression can exhibit a resistance to HF-induced obesity in mice [22], [23]. Cyclin D1 inhibits adipocyte differentiation through repression of PPARγ function [24]. Furthermore, elevated expression levels of PPARγ and its target genes, CD36 and FABP4, were also observed in KO mice with an HF. PPARγ is the most important gene involved in adipocyte terminal differentiation and survival. CD36 and FABP4 exhibit terminal differentiation gene expression and participate in lipid accumulation [25], [26]. Overall, our results suggest that loss of RGS5 increases fat deposit in adipose tissue and body weight through promoting adipocyte differentiation and hypertrophy.

High circulating FFA and hyperinsulinemia induced by peripheral insulin resistance can accelerate ectopic fat deposition in the liver by increasing the delivery of FFA or stimulating de novo lipogenesis via PPARγ and SREBP-1c, which are the key regulators of fatty acid synthesis and hepatic steatosis [27], [28]. Conversely, liver fat content correlates with hepatic insulin resistance, and decreasing the hepatic lipid concentration can improve insulin sensitivity [29], [30], [31]. Under conditions of high circulating FFA and hyperinsulinemia, RGS5 deletion in obese mice significantly promoted the activation of PPARγ and SREBP-1c and led to exacerbated phenotypes, including increased liver weight and enhanced lipid droplet accumulation.

Chronic low-grade inflammation and macrophage activation are hallmarks of obesity and insulin resistance. Compared with WT controls, RGS5 KO animals exhibited elevated levels of F4/80 in adipose tissue; this F4/80 expression implied exacerbated macrophage infiltration, which is thought to be the main source of locally produced proinflammatory adipokines [32], [33]. Furthermore, in the fat of RGS5 KO mice, we observed an increased expression of MCP-1, which can recruit macrophages into adipose tissue [34], [35]. Along with MCP-1 and F4/80, we observed higher expression levels of proinflammatory cytokines, such as IL-1β, IL-6 and TNFα, which are actively secreted by macrophages or adipocytes and cause insulin resistance [36], [37]. In addition to the local inflammation in adipose tissue induced by obesity, increased inflammatory factor secretion into the circulation from fat can influence other insulin target organs, such as skeletal muscle and liver.

Skeletal muscle is a major target of insulin, and its insulin sensitivity plays an important role in energy intake and utilization. HF-induced insulin resistance is due, in part, to increases in macrophage infiltration and the local levels of TNFα and IL-6 in skeletal muscle and the subsequent deleterious effects of these cytokines on insulin signaling in muscle tissue [38], [39]. The expression levels of inflammatory mediators, including IL-1β, IL-6, TNFα, MCP-1 and F4/80, are significantly elevated in the skeletal muscle of KO mice fed an HF. We also observed higher level of TNFα and F4/80 expression in the liver. In contrast to adipose tissue, the liver undergoes an obesity-induced activation of inflammation mainly within cells of the liver, including the resident macrophage-like Kupffer cells that crucially affect insulin sensitivity [40], [41]. TNFα can induce hepatic lipogenesis and increase hepatic triglyceride production and TNFα deficiency protected from HF-induced hepatic steatosis and insulin resistance [42], [43]. Furthermore, RGS5 deletion increased the activation of both the JNK and NF-κB pathways in the adipose tissue, liver and skeletal muscle of obese mice, which are the two critical signaling pathways of inflammation induced by obesity [44].

Notably, compared with lean animals, the excess of visceral adipose tissue and hypertrophic adipocytes in obese animals led to an increased release of adipokines, except for adiponectin, into the circulation that may be responsible for metabolic dysfunction and insulin resistance in peripheral organs, including liver and skeletal muscle [45], [46], [47]. RGS5 deletion exacerbated the pathological secretion of leptin, resistin and adiponectin from adipose tissue in obese mice.

Few studies were reported to explore the exact mechanism of RGS proteins on insulin signaling except for some researches described the phenotype of body weight and insulin sensitivity and explained the potential reasons through GPCRs and G proteins [8], [9]. Gαi2 and Gaq/11 are positive regulator of insulin action, including insulin-stimulated tyrosine phosphorylation of insulin-receptor (IR) and IR substrate 1(IRS1) and subsequent glucose uptake [6], [48], [49], [50]. However, it is well known that obese animals generally exhibit a decrease in insulin sensitivity whereas lower body weight is often accompanied by increased insulin action. So it is not surprising that the RGS5 KO mice showed weakened insulin sensitivity because obesity and obesity associated higher levels of lipids and inflammation in circulation and tissues can directly and indirectly destroy insulin signaling in multiple sites [2].

Although the present results clearly indicate RGS5 is an important protein in obesity and insulin resistance and also point to several possible mechanisms, there are some questions remain unknown and require further study, especially the reasons of increased food intake and energy consumption. Obesity is the result of an imbalance between energy intake and energy consumption. Although fatty acid oxidation is strengthened in insulin-sensitive organs, including adipose tissue and skeletal muscle in KO mice fed an HF, increased obesity and body weight maybe associated with the increased food intake induced by RGS5 deletion. G protein-mediated signaling pathways involve in regulating general behavioral functions and metabolic processes through GPCRs which widespread distribute in the body. In the neurons of hypothalamus and brain stem, a variety of GPCRs, including the melanocortins system, the NPY receptors, the cannabinoid system, the ghrelin system, the monoamine GPCRs, orexin system and the galanin receptors, can recognize neuropeptides and neurotransmitters to regulate food intake and metabolic rate [51]. RGS5 is ubiquitous in peripheral tissues and also present in brain [52]. It is not known whether this phenotype is due to the lack of expression of RGS5 in peripheral tissues or in the central nervous system, but as the function of RGS5, enhanced Gαi- and Gαq-mediated signaling pathways might contribute to the model we observed. In addition, Cho H showed that RGS5 deficiency resulted in a lean phenotype [53]. Although we can not determine the exact reasons for the discrepancies between Cho's study and ours, the phenotype of obesity in RGS5 KO mice are evident in our experiment and KO mice exhibited an increased body weight through increasing calorie intake, promoting adipocyte differentiation and hypertrophy in the adipose tissue and accelerating ectopic fat deposition in the liver. Our observation may be confirmed indirectly by Caroline's research for RGS2 [9]. RGS2 is unique among RGS proteins and limits signals mediated via Gαs- or Gαq- but not Gαi-coupled GPCRs. RGS2 KO mice exhibited a resistant to age-related or high-fat induced body weight gain, impaired adipocyte differentiation and hypertrophy comparing with WT littermates.

In summary, the present study reveals that genetic deletion of RGS5 leads to decreased insulin sensitivity induced by a high-fat diet as well as obesity, severe hepatic steatosis and inflammation, which are associated with activation of inflammatory signaling in the adipose tissue, liver and Skeletal muscle. Our findings the first demonstrate that RGS5 plays a critical role in the metabolic regulation of the body and maybe an important treatment target to obesity in the future.

## References

[1] Flegal KM, Carroll MD, Ogden CL, Curtin LR (2010). Prevalence and trends in obesity among US adults, 1999–2008.. JAMA.

[2] Gregor MF, Hotamisligil GS (2011). Inflammatory mechanisms in obesity.. Annu Rev Immunol.

[3] Wang Y, Mi J, Shan XY, Wang QJ, Ge KY (2007). Is China facing an obesity epidemic and the consequences? The trends in obesity and chronic disease in China.. Int J Obes (Lond).

[4] Yang W, Lu J, Weng J, Jia W, Ji L (2010). Prevalence of diabetes among men and women in China.. N Engl J Med.

[5] Wei H, Ahn S, Shenoy SK, Karnik SS, Hunyady L (2003). Independent beta-arrestin 2 and G protein-mediated pathways for angiotensin II activation of extracellular signal-regulated kinases 1 and 2.. Proc Natl Acad Sci U S A.

[6] Moxham CM, Malbon CC (1996). Insulin action impaired by deficiency of the G-protein subunit G ialpha2.. Nature.

[7] Huang XP, Song X, Wang HY, Malbon CC (2002). Targeted expression of activated Q227L G(alpha)(s) in vivo.. Am J Physiol Cell Physiol.

[8] Huang X, Charbeneau RA, Fu Y, Kaur K, Gerin I (2008). Resistance to diet-induced obesity and improved insulin sensitivity in mice with a regulator of G protein signaling-insensitive G184S Gnai2 allele.. Diabetes.

[9] Nunn C, Zhao P, Zou MX, Summers K, Guglielmo CG (2011). Resistance to age-related, normal body weight gain in RGS2 deficient mice.. Cell Signal.

[10] Li H, He C, Feng J, Zhang Y, Tang Q (2010). Regulator of G protein signaling 5 protects against cardiac hypertrophy and fibrosis during biomechanical stress of pressure overload.. Proc Natl Acad Sci U S A.

[11] Takata Y, Liu J, Yin F, Collins AR, Lyon CJ (2008). PPARdelta-mediated antiinflammatory mechanisms inhibit angiotensin II-accelerated atherosclerosis.. Proc Natl Acad Sci U S A.

[12] Hamzah J, Jugold M, Kiessling F, Rigby P, Manzur M (2008). Vascular normalization in Rgs5-deficient tumours promotes immune destruction.. Nature.

[13] Gunaje JJ, Bahrami AJ, Schwartz SM, Daum G, Mahoney WM (2011). PDGF-dependent regulation of regulator of G protein signaling-5 expression and vascular smooth muscle cell functionality.. Am J Physiol Cell Physiol.

[14] Hajer GR, van HTW, Visseren FL (2008). Adipose tissue dysfunction in obesity, diabetes, and vascular diseases.. Eur Heart J.

[15] Stumvoll M, Goldstein BJ, van HTW (2005). Type 2 diabetes: principles of pathogenesis and therapy.. Lancet.

[16] Tunaru S, Kero J, Schaub A, Wufka C, Blaukat A (2003). PUMA-G and HM74 are receptors for nicotinic acid and mediate its anti-lipolytic effect.. Nat Med.

[17] Kather H, Bieger W, Michel G, Aktories K, Jakobs KH (1985). Human fat cell lipolysis is primarily regulated by inhibitory modulators acting through distinct mechanisms.. J Clin Invest.

[18] Holm C (2003). Molecular mechanisms regulating hormone-sensitive lipase and lipolysis.. Biochem Soc Trans.

[19] Zimmermann R, Strauss JG, Haemmerle G, Schoiswohl G, Birner-Gruenberger R (2004). Fat mobilization in adipose tissue is promoted by adipose triglyceride lipase.. Science.

[20] Karpe F, Tan GD (2005). Adipose tissue function in the insulin-resistance syndrome.. Biochem Soc Trans.

[21] Coppack SW, Evans RD, Fisher RM, Frayn KN, Gibbons GF (1992). Adipose tissue metabolism in obesity: lipase action in vivo before and after a mixed meal.. Metabolism.

[22] Wang Y, Kim KA, Kim JH, Sul HS (2006). Pref-1, a preadipocyte secreted factor that inhibits adipogenesis.. J Nutr.

[23] Villena JA, Choi CS, Wang Y, Kim S, Hwang YJ (2008). Resistance to high-fat diet-induced obesity but exacerbated insulin resistance in mice overexpressing preadipocyte factor-1 (Pref-1): a new model of partial lipodystrophy.. Diabetes.

[24] Fu M, Rao M, Bouras T, Wang C, Wu K (2005). Cyclin D1 inhibits peroxisome proliferator-activated receptor gamma-mediated adipogenesis through histone deacetylase recruitment.. J Biol Chem.

[25] Imai T, Takakuwa R, Marchand S, Dentz E, Bornert JM (2004). Peroxisome proliferator-activated receptor gamma is required in mature white and brown adipocytes for their survival in the mouse.. Proc Natl Acad Sci U S A.

[26] Eguchi J, Yan QW, Schones DE, Kamal M, Hsu CH (2008). Interferon regulatory factors are transcriptional regulators of adipogenesis.. Cell Metab.

[27] Ahmed MH, Byrne CD (2007). Modulation of sterol regulatory element binding proteins (SREBPs) as potential treatments for non-alcoholic fatty liver disease (NAFLD).. Drug Discov Today.

[28] Schadinger SE, Bucher NL, Schreiber BM, Farmer SR (2005). PPARgamma2 regulates lipogenesis and lipid accumulation in steatotic hepatocytes.. Am J Physiol Endocrinol Metab.

[29] Savage DB, Choi CS, Samuel VT, Liu ZX, Zhang D (2006). Reversal of diet-induced hepatic steatosis and hepatic insulin resistance by antisense oligonucleotide inhibitors of acetyl-CoA carboxylases 1 and 2.. J Clin Invest.

[30] Dentin R, Benhamed F, Hainault I, Fauveau V, Foufelle F (2006). Liver-specific inhibition of ChREBP improves hepatic steatosis and insulin resistance in ob/ob mice.. Diabetes.

[31] Neschen S, Morino K, Hammond LE, Zhang D, Liu ZX (2005). Prevention of hepatic steatosis and hepatic insulin resistance in mitochondrial acyl-CoA:glycerol-sn-3-phosphate acyltransferase 1 knockout mice.. Cell Metab.

[32] Fain JN (2006). Release of interleukins and other inflammatory cytokines by human adipose tissue is enhanced in obesity and primarily due to the nonfat cells.. Vitam Horm.

[33] Weisberg SP, McCann D, Desai M, Rosenbaum M, Leibel RL (2003). Obesity is associated with macrophage accumulation in adipose tissue.. J Clin Invest.

[34] Lumeng CN, Deyoung SM, Bodzin JL, Saltiel AR (2007). Increased inflammatory properties of adipose tissue macrophages recruited during diet-induced obesity.. Diabetes.

[35] Kirk EA, Sagawa ZK, McDonald TO, O'Brien KD, Heinecke JW (2008). Monocyte chemoattractant protein deficiency fails to restrain macrophage infiltration into adipose tissue [corrected].. Diabetes.

[36] Hotamisligil GS, Shargill NS, Spiegelman BM (1993). Adipose expression of tumor necrosis factor-alpha: direct role in obesity-linked insulin resistance.. Science.

[37] Kern PA, Ranganathan S, Li C, Wood L, Ranganathan G (2001). Adipose tissue tumor necrosis factor and interleukin-6 expression in human obesity and insulin resistance.. Am J Physiol Endocrinol Metab.

[38] Hong EG, Ko HJ, Cho YR, Kim HJ, Ma Z (2009). Interleukin-10 prevents diet-induced insulin resistance by attenuating macrophage and cytokine response in skeletal muscle.. Diabetes.

[39] Steinberg GR, Michell BJ, van DBJ, Watt MJ, Carey AL (2006). Tumor necrosis factor alpha-induced skeletal muscle insulin resistance involves suppression of AMP-kinase signaling.. Cell Metab.

[40] Baffy G (2009). Kupffer cells in non-alcoholic fatty liver disease: the emerging view.. J Hepatol.

[41] Odegaard JI, Ricardo-Gonzalez RR, Red EA, Vats D, Morel CR (2008). Alternative M2 activation of Kupffer cells by PPARdelta ameliorates obesity-induced insulin resistance.. Cell Metab.

[42] Feingold KR, Grunfeld C (1987). Tumor necrosis factor-alpha stimulates hepatic lipogenesis in the rat in vivo.. J Clin Invest.

[43] De Taeye BM, Novitskaya T, McGuinness OP, Gleaves L, Medda M (2007). Macrophage TNF-alpha contributes to insulin resistance and hepatic steatosis in diet-induced obesity.. Am J Physiol Endocrinol Metab.

[44] Donath MY, Shoelson SE (2011). Type 2 diabetes as an inflammatory disease.. Nat Rev Immunol.

[45] Rosen ED, Spiegelman BM (2006). Adipocytes as regulators of energy balance and glucose homeostasis.. Nature.

[46] Guilherme A, Virbasius JV, Puri V, Czech MP (2008). Adipocyte dysfunctions linking obesity to insulin resistance and type 2 diabetes.. Nat Rev Mol Cell Biol.

[47] Rabe K, Lehrke M, Parhofer KG, Broedl UC (2008). Adipokines and insulin resistance.. Mol Med.

[48] Kreuzer J, Nurnberg B, Krieger-Brauer HI (2004). Ligand-dependent autophosphorylation of the insulin receptor is positively regulated by Gi-proteins.. Biochem J.

[49] Kanoh Y, Ishizuka T, Morita H, Ishizawa M, Miura A (2000). Effect of pertussis toxin on insulin-induced signal transduction in rat adipocytes and soleus muscles.. Cell Signal.

[50] Usui I, Imamura T, Satoh H, Huang J, Babendure JL (2004). GRK2 is an endogenous protein inhibitor of the insulin signaling pathway for glucose transport stimulation.. EMBO J.

[51] Schioth HB (2006). G protein-coupled receptors in regulation of body weight.. CNS Neurol Disord Drug Targets.

[52] Larminie C, Murdock P, Walhin JP, Duckworth M, Blumer KJ (2004). Selective expression of regulators of G-protein signaling (RGS) in the human central nervous system.. Brain Res Mol Brain Res.

[53] Cho H, Park C, Hwang IY, Han SB, Schimel D (2008). Rgs5 targeting leads to chronic low blood pressure and a lean body habitus.. Mol Cell Biol.

